# Scopolamin-Morphin Anesthesia

**Published:** 1905-12

**Authors:** 


### Scopolamin-Morphin Anesthesia.

If the reports of the results of this method of anesthesia are confirmed from many sources, it should prove to be the ideal anesthetic for general use. Since its introduction in Europe, in 1900, it has been used chiefly on the continent. Ries, of Chicago, reports his experience with it in over one hundred cases, in *Surgery, Gynecology and Obstetrics,* for October. His formula is, one-half grain morphin mixed in water with one-fiftieth grain scopolamin hydrobromate, which is divided into three doses, to be injected hypodermically. One dose is given two and one-half, one dose one and one-half, and one dose one-half hour before the time set for operation. After the first dose patient becomes drowsy, sleeps soundly after the second and is insensible to pain after the third dose. In about one-half the cases prolonged operations are conducted without consciousness on part of the patient. If he is partly conscious, an extremely small amount of chloroform or ether is necessary. The advantages of this procedure are the absence of the usual worry and excitement preceding general anesthesia and disagreeable sensations, with nausea and vomiting, following the operation, since the patient sleeps about five hours after the last injection.

Ries has not used it on children under 12 years, but has found it invaluable in old and decrepit patients, in whom as low as one-third of the mentioned dose is sufficient. He states there is no record of a human being having been killed by scopolamin and no case on record of a death after operation that could be charged to scopolamin-morphin anesthesia.

It has been used extensively in Europe for obstetrical cases. One-third of the given dose is administered every five or six hours and renders the labor practically painless. When necessary, obstetrical operations can be performed under its influence or by addition of a small amount of chloroform.—*N. W. Medicine.*

